# Transcriptome of Chicken Liver Tissues Reveals the Candidate Genes and Pathways Responsible for Adaptation into Two Different Climatic Conditions

**DOI:** 10.3390/ani9121076

**Published:** 2019-12-03

**Authors:** Himansu Kumar, Asankadyr U. Iskender, Krishnamoorthy Srikanth, Hana Kim, Asankadyr T. Zhunushov, Hyojun Chooq, Gul Won Jang, Youngjo Lim, Ki Duk Song, Jong Eun Park

**Affiliations:** 1 Division of Animal Genomics and Bioinformatics, National Institute of Animal Science, Wanju 55365, Korea; himanshu.genetics@gmail.com (H.K.); sacredtkd@gmail.com (A.U.I.); kris87@korea.kr (K.S.); hanakim0307@gmail.com (H.K.); hyojy@korea.kr (H.C.); kwchang@korea.kr (G.W.J.); topair707@korea.kr (Y.L.); 2 Institute of Biotechnology, National Academy of Science of Kyrgyzstan, Bishkek, 720071, Kyrgyzstan; junushov@mail.ru; 3 The Animal Molecular Genetics and Breeding Center, Department of Animal Biotechnology, JeonBuk National University, Jeonju 54896, Korea; kiduk.song@gmail.com

**Keywords:** Hanhyup chicken, transcriptome, liver tissues, PPAR pathway

## Abstract

**Simple summary:**

In this study, RNA-Seq data between liver tissues of Hanhyup chicken maintained in Korea and Kyrgyzstan was compared. We then investigated the candidate genes involved in response to environmental changes such as altitude, humidity, temperature etc. We found that 315 genes were differentially expressed genes (DEGs) in the Kyrgyz environment, out of which 174 and 141 were up- and down-regulated respectively. GO and KEGG pathway enriched between the two climatic conditions have been investigated. The identified candidate DEGs and enriched pathways may involve in the acclimatization of Hanhyup chicken to the Kyrgyz environment.

**Abstract:**

RNA sequencing was used to profile the liver transcriptome of a Korean commercial chicken (Hanhyup) at two different environments (Korea and Kyrgyzstan) to investigate their role during acclimatization into different climatic conditions. Ten samples from each location were analyzed to identify candidate genes that respond to environmental changes such as altitude, humidity, temperature, etc. Sequencing reads were preprocessed, aligned with the reference genome, assembled and expressions were estimated through bioinformatics approaches. At a false discovery rate (FDR) <0.05 and fold change (FC) ≥2, we found 315 genes were DE. Out of 315 DE genes, 174 and 141 were up- and down-regulated respectively in the Kyrgyz environment. Gene ontology (GO) enrichment analysis showed that the differentially expressed genes (DEGs) were associated with energy metabolism such as pyruvate and lactate metabolic processes, and glycerol catabolic process. Similarly, KEGG pathway analysis indicated pyruvate metabolism, glycolysis/gluconeogenesis, biosynthesis, citrate cycles were differentially enriched in the Kyrgyz environment. DEGs like *TSKU, VTG1, SGK, CDK2,* etc. in such pathways are highly involved in the adaptation of organisms into diverse climatic conditions. Our investigation may serve as a resource for the chicken industry, especially in exporting Hanhyup chicken from Korea to other countries.

## 1. Introduction

The consumption of chicken meat has increased worldwide in recent years because of its quality, taste, and rich nutritious content [1,2]. Demand for Hanhyup chicken also increased due to its cheaper price compared to other animal proteins such as beef and pork [3]. Hanhyup chicken is a commercial Korean native chicken raised to fulfill the meat consumption of Korea [4]. According to USDA foreign agricultural service report 2018, it has been reported that Korea’s chicken production has increased from 910,000 to 932,000 metric tons (accessed on 15 September 2019, web page: https://www.fas.usda.gov/data/south-korea-poultry-and-products-annual-3). Due to the increased demand for processed chicken meat, Korea’s chicken exports increased up to 29,000 metric tons by 2018, mainly to Vietnam, Japan, China, and Kyrgyzstan (GAIN Report Number: KS1832). Adaptation of Hanhyup chicken to different climatic conditions is needed to enhance local production of Hanhyup chicken in these countries, which will support local farmers and the regional economy.

Animals have evolved mechanisms to transit and survive under different climatic conditions, and liver tissues plays a central role in adaptive response [5]. It is reported that the liver helps the organism to combat environmental changes such as fluctuations in the level of oxygen due to altitudinal differences, temperature changes etc. [6]. Liver tissues are not only comprised of hepatocytes cells but also composed of many blood and immune cells [7]. Several studies reported that multiple pathways such as gluconeogenesis, fatty acids metabolism, PPAR pathways are differentially regulated during adaptation [5,8,9,10]. Identification of such pathways and genes involved in adaptation to two different environmental conditions may help in elucidation of the molecular mechanism behind such adaptation [11].

A previous study had reported that cold-inducible RNA-binding protein (CIRBP) gene play a key role against the cold [12]. It may act as a universal marker for cold exposure in all vertebrates liver tissues as well as in other tissues [13]. Baze et al., in 2010 reported the role of liver tissues in acclimatization and adaptation under hypoxia conditions in mammals [14]. They found genes such as transferrin, *VEGF, GAPDH* were differentially enriched in response to hypoxia. Some pathways like Hypoxia-inducible factor (HIF) was significant in both acute and chronic hypoxia condition [7]. Sixin Liu et al., in 2014 studied the transcriptome of Rainbow Trout liver tissues and reported some of the genes and pathways handling the stresses [15]. MHC class II alpha chain (g17813.1), heat shock protein (g03437.1), neurofilament light polypeptide (g07619.1) were top upregulated, and tsc22 domain member 3, (g00766.1), barrier to auto integration factor (g05419.1), transcription factor cp2-like 1 (g39048) genes were top down-regulated genes responsible for adaptation in stress [15]. Transcriptome analysis in the liver tissues of *Coilia nasus*, Du et al., in 2014 identified significantly stress-related pathways such as metabolism (glucose and lipid) and immunity [16]. They also reported liver injury indicators such as Alanine aminotransferase (ALT), and aspartate transaminase (AST) were differentially abundant during stress and induces liver injury [16]. 

Cobel et al., 2014 studied the transcriptome response of broiler chicken liver to temperature variation [17]. They found pathways such as cell signaling and endocrine system development and function to be differentially enriched in response to ambient temperature variation. In addition, transcriptome analysis also revealed that high ambient temperature causes perturbation in calcium levels [17]. Singh, et al. in 2018 reported the adaptation response against the environmental changes of migratory birds through transcriptome analysis [18]. Trefts et al., 2015 and Kurauti et al., 2016 reported that the liver plays a crucial role in metabolic homeostasis, such as glucose and lipid metabolism. Liver stores the nutrient substrates for use of other tissue during the transition from one environment to another [19].

In this study, an effort was made to establish an understanding of the molecular mechanism of Hanhyup chicken liver tissues and their involvement during adaptation into the new environment. We tried to decipher the pathways and genes involved in the adaptation of the Hanhyup chicken to the Kyrgyz environment and identified candidate markers through transcriptome analysis. 

## 2. Materials and Methods 

### 2.1. Ethics Statement and Sample Collection

Geographical location and a huge difference in climatic conditions throughout the year make Korea and Kyrgyzstan incomparable countries. There exists a vast difference in altitude, with Korea located at 250 m above mean sea level and Kyrgyzstan at 2500 m above mean sea level. The average humidity in Korea is 70% while in Kyrgyzstan it’s about 40%. The Hanhyup chicken breed was reared according to commercial parameters, in NIAS campus rearing facility in South Korea. The summer season was considered for sampling of chickens. We selected two months old healthy female chickens by providing the same nutrition to all animals as guided by Hanhyup breeder a Korean Poultry breeding company. The same breed of chickens was reared under similar guidelines at the Institute of Biotechnology, National Academy of Science of Kyrgyz Republic. Liver tissues from all ten samples from each group were dissected and stored at −80 °C for RNA extraction. All animal experiments were performed by following the protocols approved by the approval committee of the National Institute of Animal Science (NIAS), Rural Development Administration (RDA), South Korea under the approval number 2018-262.

### 2.2. RNA Extraction, Library Construction, and Sequencing

RNA from each liver tissues was isolated using the Trizol/chloroform/isopropanol method following the manufacturer’s protocol (Invitrogen^TM^, Carlsbad, CA, USA) was used to extract. The nano photometer (IMPLEN, CA, USA), was used to check the purity and integrity of RNA. The quality of the isolated RNA was measured by Bioanalyzer 2100 system using RNA Nano 6000 Assay kit (Agilent Technologies, CA, USA). Samples with a RIN (RNA integrity number) value >8 were considered for library preparation. The library was prepared by random fragmentation of sample, ligation of adapter, and tagmentation with the help of NEBNext Ultra™ RNA Library Prep Kit for Illumina (New England Biolabs (NEB), Ipswich, MA, USA) by following manufacturers protocol. Obtained adapter-ligated fragments were then amplified by PCR and gel purification was performed. TruSeq PE cluster kit v4-cBot-HS (Illumia) was used to generate the clusters as per the guidelines provided by the manufacturer. The library was loaded into flow cell of Illumina HiSeq 2500 platform for sequencing and generated the paired-end reads. Details of reads of each sample are shown in Supplementary Table S1. The real-time analysis (RTA) software was used for base calling of illlumina sequencer generated raw images. The Illumina package bcl2fastq was used to convert base call to FASTQ reads. 

### 2.3. Quality Analysis and Mapping of Reads

The FASTQC program was used to check the quality of the obtained raw reads (Andrews S (2010) FastQC: https://www.bioinformatics.babraham.ac.uk/projects/fastqc/. Accessed 2019 May 6). Good quality reads were obtained by removing the low-quality reads, poly-N reads, and phred-score ≤20. The Q20, Q30, and GC% were calculated. Trimmomatic (v0.33) was used to remove adapters from 5’ and 3’ end [20]. Subsequently, filtered sequences were used for reference-based genome mapping. To assemble the reads, clean reads were mapped against the reference genome of chicken (galGal6) with the help of Tophat2 (v2.1.1) mapping program [21]. Cufflink and cuffdiff (v2.2.1) suites were used to estimate the fragments per kilobase million (FPKM) value of each transcript based on expression value count [22]. 

### 2.4. Differential Gene Expression Analysis

ExDEGA (v.1.6.5) an excel based differentially expressed gene analysis tool, developed by ebiogen, South Korea, was used to estimate the DEGs. The p-values and FDR were calculated and assigned to each gene. Genes with fold change ≥2 and FDR < 0.05, were considered as DE genes. 

### 2.5. GO, KEGG Pathway Analysis, and Network Representation

List of up- and down-regulated DEGs, were subjected to DAVID online tool for gene enrichment analysis with fold change 2, and FDR < 0.05) [23]. The Benjamini–Hochberg FDR method was used to determine the significance of enrichment. Background species: Gallus gallus, level of GO: GOTERM_BP, CC, MF_DIRECT (the categories denote DAVID defined defaults) were selected. Gene Ontology database was used to establish the association of gene products with DE genes in terms of molecular function, cellular component, and biological processes (GO: http://www.geneontology.org/) [24]. Further, to know the relation of differentially expressed genes with metabolic pathways, we used Kyoto Encyclopedia of Genes and Genomes Pathway (KEGG) database (http://www.genome.jp/kegg) pathway database [25,26]. The top ten enriched pathways related genes were used for the construction of a biological network with the help of Cytoscape (v3.2.1) and plugin ClueGO [27,28].

## 3. Results

### 3.1. Raw Reads Quality, Sequencing and Mapping

With the help of the Illumina HiSeq 2500 platform, we found 7–10 million reads. GC percentage and Q20/Q30 ranged between 44.9–47.5% and 92–98% respectively, as shown in Supplementary Table S1. TopHat2 alignment tool was used to map 92–95% sequences with the reference genome of chicken (galGal6). 

### 3.2. RNA-Seq of Liver Tissue

**Figure 1:**
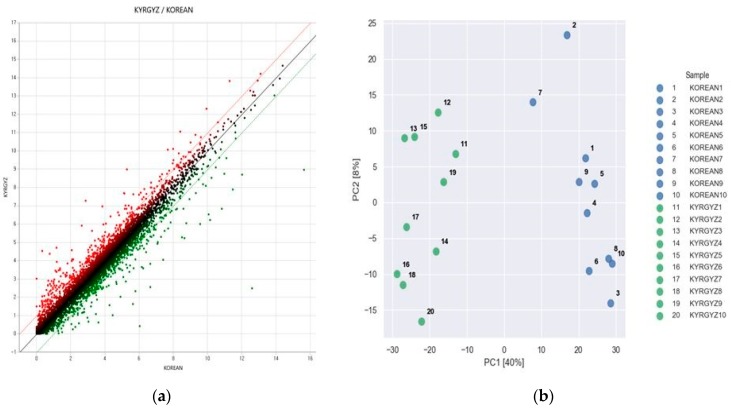
(**a**) Scatter plot between Korean and Kirgizstan chicken tissue sample, (**b**) principal component analysis (PCA) of all samples.

Cufflink suite was used to estimate the genetic expression of transcripts. The transcripts with ≥1 FPKM value were used to generate a scatter plot between Korean and Kyrgyzstan chicken liver tissues as shown in Figure 1a. The red and green dots are representations of up- and down-regulated genes respectively, whereas the black one indicate non-DEGs. Gene expression levels were estimated by using FPKM values (Roberts et al., 2011). The FPKM values were normalized based on the quantile normalization method using EdgeR (R Development Core Team, 2016) [29,30,31]. As shown in Figure 1a, the horizontal and vertical coordinates of the scatter plot are the expression level in Korea and Kyrgyzstan respectively. Principal component analysis (PCA) using DEGs indicated a clear separation of chicken raised in Korea and Kyrgyzstan (Figure 1b).

**Figure 2:**
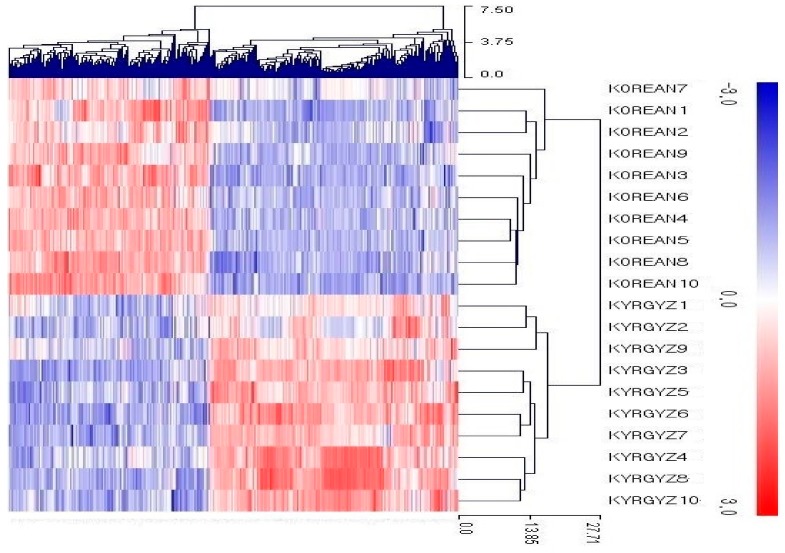
Hierarchical clustering of DEGs in Kyrgyz environment, column represents the gene expression pattern of each sample. Up- and down-regulated transcripts are shown in red and blue color respectively.

Normalized read counts were used for differential expression analysis between Korean and Kyrgyz environement, by considering genes with fold change 2 and FDR < 0.05. We found 174 and 141 up- and down-regulated genes respectively. The hierarchical clustering method in MeV v4.9 tool was used for clustering genes based on Euclidean distance of z-score and average linkage [32]. Heat map along with the clustering results are shown in Figure 2. Sample wise transcriptome expression results with p-value are provided in the Supplementary File (Excel sheet 1 and 2).

### 3.3. GO Enrichment and KEGG Pathways Analysis

**Figure 3:**
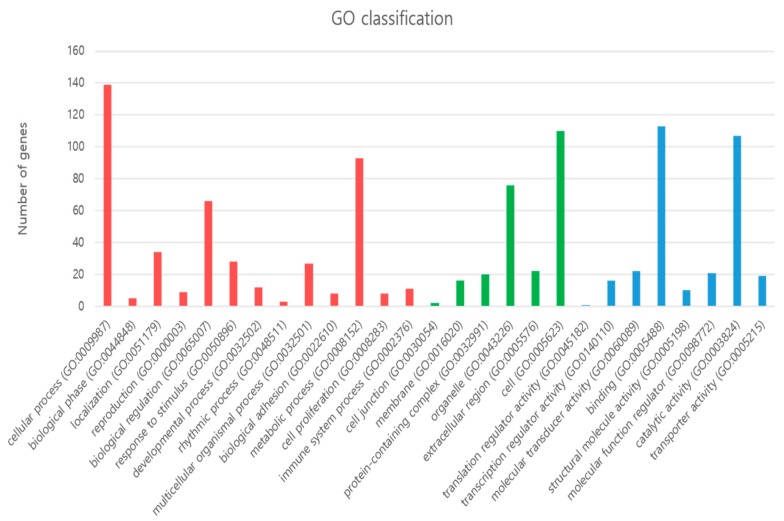
Significantly enriched gene ontology terms; such as biological processes, cellular components, and molecular functions are shown in red, green, and blue color respectively.

GO was used for the functional annotation of DEGs (Figure 3). On the basis of significant enrichment (FDR < 0.05), 13 biological processes were found differentially enriched and related to following processes such as: cellular (GO: 0009987) metabolic processes (GO: 0008152), biological regulation (GO: 0065007), cellular localization (GO: 0051179), multicellular organismal processes (GO: 0032502), developmental processes (GO: 0032502) etc. Under cellular components, we found six GO terms were significantly enriched including cellular (GO: 0005623), extracellular regions (GO: 0005576), organelle (GO: 0043226), protein-containing complex (GO: 0032991), membrane (GO: 0016020) and cellular junctions (GO: 0030054). Similarly, under molecular function, we found seven GO terms were significantly enriched including catalytic activity (GO: 0003824) and binding activity (GO: 0005488) as shown in Figure 3 and Supplementary Table (Table S2a).

**Figure 4:**
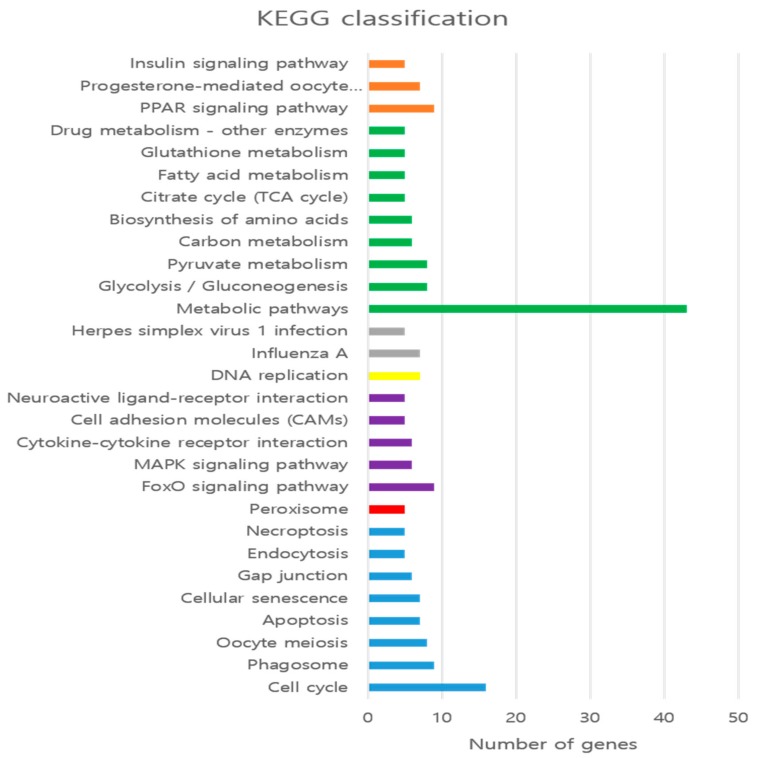
KEGG pathways enrichment analysis of DEGs (*p* < 0.05) obtained from RNA-seq results. Pathways are shown according to the number of DEGs.

To elucidate the biological functions of DEGs and their molecular interaction in the cells, we performed the biological pathway analysis. We identified 30 enriched KEGG pathways (*p* < 0.05). KEGG pathways clustered into seven groups such as cellular processes (blue), drug development (red), environmental information (purple), genetic information (yellow), human disease (grey), metabolism (green), and organismal systems (orange) as shown in Figure 4. Some of the highly enriched pathways include metabolic pathways, cell cycle, glycolysis/gluconeogenesis, pyruvate metabolism, PPAR signaling pathway, MAPK signaling pathway, FoxO signaling pathway as shown in Figure 4 and Supplementary File (Table S3).

### 3.4. Network Representation of Top Ten KEGG Pathways

**Figure 5:**
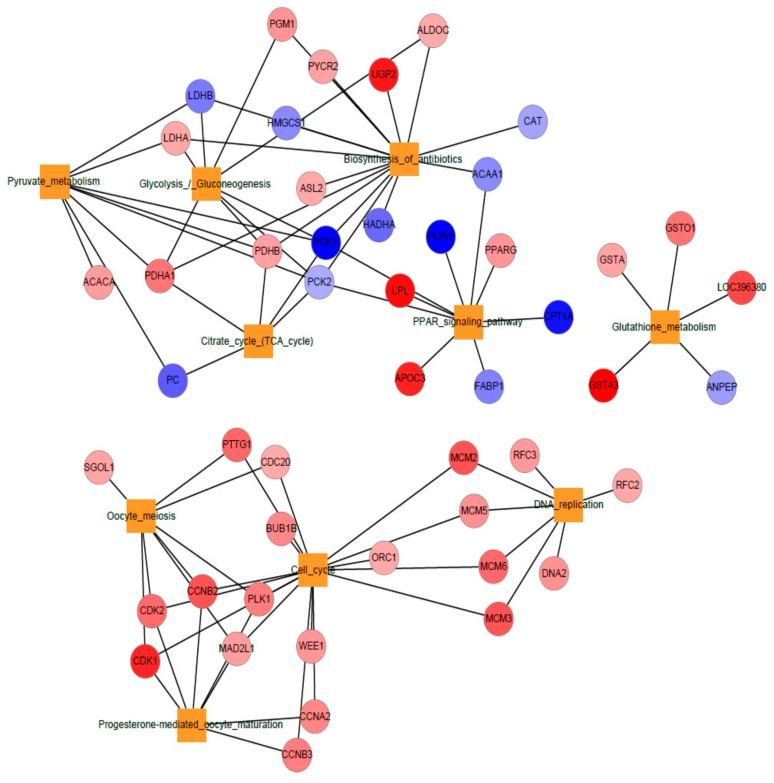
Top ten enriched KEGG pathways network, color gradient from red to blue indicates the up -and down-regulated genes respectively.

We selected the top 10 KEGG pathways which are differentially enriched, for network representation by a hypergeometric test with Benjamini–Hochberg FDR correction (fold change 2, FDR < 0.05). The regulations of the genes involved in pathways are shown as a color gradient from red to blue indicates, up- or down-regulation, respectively. Pathways such as cell cycle, oocyte meiosis, progesterone mediated oocyte maturation, DNA replication, and glutathione metabolism, etc. having only upregulated genes. Whereas, pathways like Pyruvate metabolism, glycolysis/gluconeogenesis, biosynthesis of antibiotics and citrate cycle are having up- and down-regulated genes both (Figure 5).

## 4. Discussion

In the present study, we performed a transcriptome analysis to decipher the changes in liver transcriptome response in the liver tissue of Hanhyup chicken introduced to Kyrgyzstan compared to those raised in Korea. The difference in average altitude, humidity, temperature etc. between the countries can impact the genetic expression of liver tissue of chicken during adaptation to the different climatic conditions. A total of 315 DE genes were identified in transcriptome analysis. On the basis of differential gene expression analysis of liver tissues, the top upregulated genes are *TSKU, PDXK, GSTA3, SERPINB10, ANKRD22, S100A9, LYG2* and top down-regulated genes are *VTG1, APOV1, PCK1, HMGCL*.

Gene ontology and KEGG pathway analysis of these genes showed that 27 GO terms and 30 KEGG pathways were enriched. It also showed that the enriched genes were mainly involved in pyruvate metabolism, glycolysis/gluconeogenesis, biosynthesis of antibiotics, PPAR, glutathione metabolism, and citrate cycle pathways (*p* < 0.05). Pyruvate metabolism pathway plays an important role in the synthesis of glucose from lactate and dihydroxyacetone in chicken liver [33]. Many studies have reported that under any kind of stress, the chicken liver plays a significant role in maintaining homeostasis through glycolysis/Gluconeogenesis pathways [17,34,35]. Glucose is an essential nutrient for any tissue, and the liver provides glucose through gluconeogenesis [36]. In our study, enrichment of such pathway suggesting that the glucose level might have been modulated through gluconeogenesis during Hanhyup chicken acclimation to the Kyrgyz environment [37].

PPAR, a critical pathway in lipid metabolism was significantly enriched. Three genes were up-regulated *(PPAR γ, Apc-CIII, LPL,)* and six were down-regulated *(FABP, FABP1, Thiolase B, PEPCK, CPT-1, Perilipin)* in the PPAR pathway [36]. *PPARγ* gene acts as a regulator of adipocyte differentiation and is involved in multiple diseases such as obesity, diabetes, atherosclerosis, and cancer. The up-regulation of *PPARγ* gene in adipose tissue and the brain has been reported to be associated with stress-related behavior [38]. Overrepresentation of *LPL*, results in the deposition of lipid on the arterial wall [39]. It has been reported that the *LPL* gene is weakly expressed in the liver, but up-regulated when carbohydrate availability is less. It facilitates the generation of ketone into the liver and subsequently provides energy to the brain and muscle [40]. The down-regulated gene family FABP is involved in the regulation of inflammation and cellular metabolism via the PPAR pathway. FABP1 particularly activates the *PPARγ,* and also acts as downstream transcriptional target of PPARγ. Enrichment of PPAR pathway and involvement of DE genes in this pathway indicates that during adverse conditions such as less availability of carbohydrates, diseased conditions, and stress conditions etc., PPAR helps in adaptation.

Further, biosynthesis of antibiotics pathways, responsible for the natural synthesis of antibiotics, was also enriched. The enrichment of such pathways may indicate the defense against the stress during acclimatization in a new environment [41]. DEGs in these pathways such as *UGP2, ALDOC, ASL2* and *ACAA1, CAT, HADHA* are up- and down-regulated respectively in Kyrgyz condition. In liver tissue, *UGP2* encodes the enzyme which can activate the carbohydrate interconversions [42]. Similarly, the gene *ACAA1* involved in lipid and fatty acids metabolism, may indicate that during a change of environmental conditions, these genes are actively involved in energy metabolic pathways to full fill the requirements of the liver tissue of chicken.

Likewise, liver is also a source of glutathione circulation, enrichment of glutathione metabolism in our study indicates the role of Hanhyup chicken liver during stress caused by environmental factors such as temperature and humidity [37]. The citrate cycle pathway is known for the oxidation of carbohydrates and fatty acids. It plays an important role in adapting to low levels of oxygen [43]. Désert, Colette, et al. in 2008 reported that the liver plays a central role during starvation in chicken [5]. Overall, we tried to pinpoint the various pathways and genes which are significant for adaptation of Hanhyup chicken to the Kyrgyz environment.

## 5. Conclusions

This study provides an insight into the genetic mechanisms involved in the adaptation of Hanhyup chicken, a Korean commercial chicken breed, to the Kyrgyz environment. The results of this study revealed that several metabolic pathways such as PPAR signaling, FoxO signaling, citrate cycle, gluconeogenesis, etc. are modulated during acclimation of Hanhyup chicken to Kyrgyz environment. Highly enriched genes such as *PPAR γ, Apc-CIII, LPL, FABP, PCK1, UGP2,* and *GSTA3* are potential candidate genes for local adaptation and acclimation. However, part of it might be caused by genetic differences and breeding conditions also. A detailed functional experiment could be carried out to validate these candidate genes. This study will be beneficial for poultry industries, especially for understanding the adaptation response of chickens from one environment to another. 

## Data Availability

Sequencing reads are submitted into NCBI under SRA submission portal with project code PRJNA577590.

## Supplementary Materials

The following are available online at https://www.mdpi.com/2076-2615/9/12/1076/s1. Table S1: Table S1. Sample wise detail description of total bases, read count, GC (%), AT (%), Q20 (%), Q30 (%). Table S2: Count_Fold_Enrichment_P_value_of_GO_KEGG. Table S3: Some of the highly enriched pathways.
