# Supplementary material for: Transcriptome of Chicken Liver Tissues Reveals the Candidate Genes and Pathways Responsible for Adaptation into Two Different Climatic Conditions
Source: Animals (Basel). 2019 Dec 3;9(12):1076. doi: 10.3390/ani9121076 (PMC6940799; doi:10.3390/ani9121076)
Supplement: Supplementary file 1 [file animals-09-01076-s001.zip › animals-620031-supplementary/Table S1. Sample wise detail description of total bases, read count, GC (%), AT (%), Q20 (%), Q30 (%)..docx]

Table S1. Sample wise detail description of total bases, read count, GC (%), AT (%), Q20 (%), Q30 (%).

| **Sample** | **Total Bases** | **Read Count** | **GC (%)** | **AT (%)** | **Q20 (%)** | **Q30 (%)** |
| --- | --- | --- | --- | --- | --- | --- |
| Korean_1 | 7,129,973,598 | 70,593,798 | 46.29 | 53.72 | 98.31 | 95.63 |
| Korean_2 | 7,715,135,884 | 76,387,484 | 46.63 | 53.37 | 98.53 | 96.08 |
| Korean_3 | 8,177,511,864 | 80,965,464 | 46.14 | 53.86 | 98.28 | 95.54 |
| Korean_4 | 8,725,152,246 | 86,387,646 | 45.59 | 54.41 | 98.27 | 95.58 |
| Korean_5 | 8,257,685,664 | 81,759,264 | 46.66 | 53.35 | 98.26 | 95.49 |
| Korean_6 | 6,523,120,148 | 64,585,348 | 46.67 | 53.33 | 98.28 | 95.54 |
| Korean_7 | 7,496,779,742 | 74,225,542 | 46.13 | 53.87 | 98.26 | 95.54 |
| Korean_8 | 8,092,116,970 | 80,119,970 | 46.36 | 53.65 | 98.38 | 95.76 |
| Korean_9 | 8,209,156,376 | 81,278,776 | 44.98 | 55.02 | 98.55 | 96.14 |
| Korean_10 | 7,041,300,648 | 69,715,848 | 45.84 | 54.16 | 98.19 | 95.36 |
| Kyrgyz_1 | 6,927,620,906 | 68,590,306 | 46.33 | 53.67 | 96.36 | 93.72 |
| Kyrgyz_2 | 7,723,479,898 | 76,470,098 | 46.06 | 53.95 | 95.94 | 93.18 |
| Kyrgyz_3 | 8,106,517,146 | 80,262,546 | 46.45 | 53.55 | 95.78 | 92.79 |
| Kyrgyz_4 | 8,332,527,674 | 82,500,274 | 47.22 | 52.78 | 97.16 | 94.98 |
| Kyrgyz_5 | 9,183,511,860 | 90,925,860 | 46.76 | 53.24 | 96.79 | 94.38 |
| Kyrgyz_6 | 9,305,458,048 | 92,133,248 | 45.33 | 54.67 | 96.25 | 93.55 |
| Kyrgyz_7 | 10,180,061,488 | 100,792,688 | 46.97 | 53.03 | 96.90 | 94.56 |
| Kyrgyz_8 | 9,925,504,320 | 98,272,320 | 47.54 | 52.46 | 96.70 | 94.25 |
| Kyrgyz_9 | 7,343,488,204 | 72,707,804 | 47.37 | 52.63 | 96.50 | 93.94 |
| Kyrgyz_10 | 10,806,444,096 | 106,994,496 | 46.44 | 53.56 | 96.61 | 94.13 |
