# Supplementary figures and images for: Transcriptome of Chicken Liver Tissues Reveals the Candidate Genes and Pathways Responsible for Adaptation into Two Different Climatic Conditions
Source: Animals (Basel). 2019 Dec 3;9(12):1076. doi: 10.3390/ani9121076 (PMC6940799; doi:10.3390/ani9121076)

**Table S2 Count_Fold_Enrichment_P_value_of_GO_KEGG**

**Table S2a**


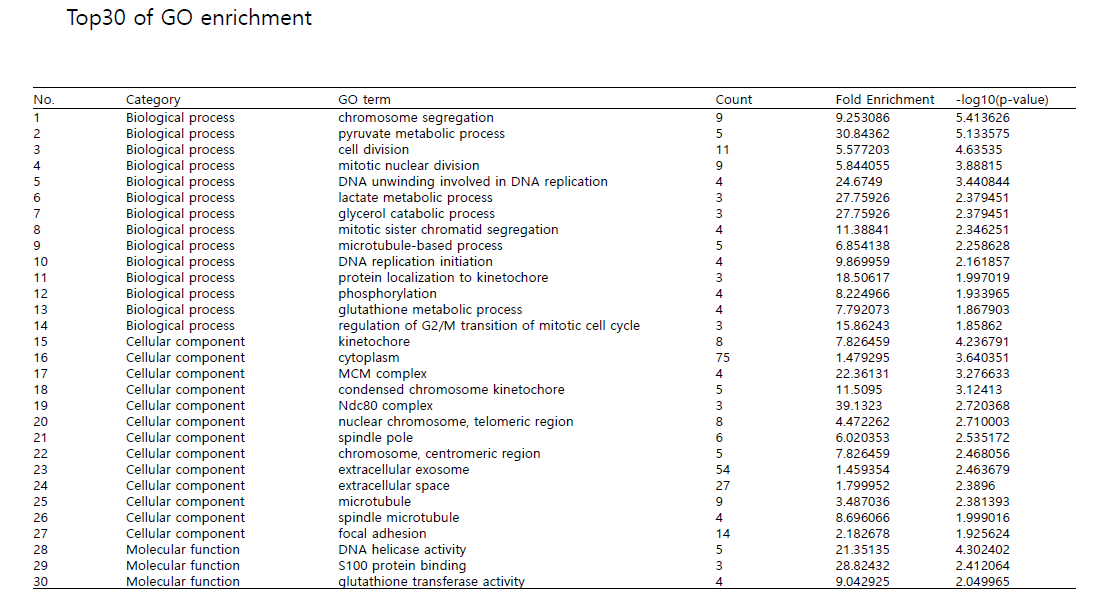


**Table S2b**


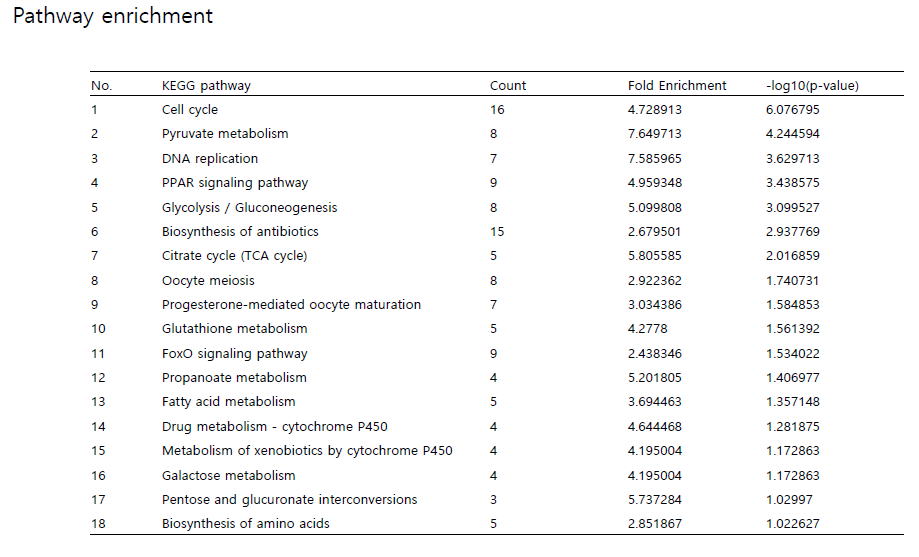

Supplement: Supplementary file 1 [file animals-09-01076-s001.zip › animals-620031-supplementary/Table S2 Count_Fold_Enrichment_P_value_of_GO_KEGG.docx]
